# Invisible lives: understanding the food insecurity and food-seeking behaviour among Bangladeshi undocumented migrants amidst the COVID-19 pandemic

**DOI:** 10.1186/s12889-025-25851-x

**Published:** 2025-12-04

**Authors:** Md. Khaled Sifullah, Md. Salman Sohel, Md. Mizanur Rahman, Noyon Ali, Mosamat Umma Kulsum, Mohammad Fakhrul Islam

**Affiliations:** 1 Department of Nutrition and Food Engineering, Daffodil International University, Dhaka, 1216 Bangladesh; 2 Faculty of Health, Education and Life Sciences, Birmingham City University, Birmingham, UK; 3 School of Management, University of Science and Technology of China, Anhui-230026, China; 4 Department of Development Studies, Daffodil International University, Dhaka, 1216 Bangladesh; 5 BRAC Business School, BRAC University, Dhaka, 1212 Bangladesh; 6 Department of Business Administration, Eastern University, Dhaka, 1216 Bangladesh; 7 Senior Manager, Friendship NGO, Baridhara North Road, Dhaka, 1212 Bangladesh; 8 Sustainability Competence Centre, Széchenyi István University, Győr, Hungary

**Keywords:** *COVID-19* pandemic, Undocumented migrants, Bangladeshi migrants, Food insecurity, Food-Seeking behavior, Vulnerable populations

## Abstract

**Background:**

Undocumented migrants often face significant socio-economic and health vulnerabilities, which are further intensified during global crises such as the COVID-19 pandemic. Among these challenges, food insecurity emerges as a critical concern, particularly for migrants lacking legal status, social protection, and access to basic services. This study examines the prevalence of food insecurity and food-seeking behaviour-related coping mechanisms among Bangladeshi undocumented migrants living in Malaysia, Iraq, and Libya during the COVID-19 pandemic.

**Methods:**

Twenty-seven undocumented Bangladeshi migrants were interviewed using a qualitative Interpretative Phenomenological Approach (IPA). The author combined an integrated data-driven inductive technique to code and analyse the data. The data analysis followed the six-step process of Interpretative Phenomenological Analysis (IPA).

**Results:**

The study reveals that COVID-19 has significantly impacted their food security, including food unavailability and inaccessibility, decreasing consumption, increasing reliance on cheaper and malnutrition food, as well as hunger and starvation. To cope, they adopted various food-seeking behaviors, such as receiving support from friends and relatives, taking loans, seeking food assistance from different sources, and selling personal belongings.

**Conclusion:**

The findings highlight the vulnerability of undocumented migrants during crises and suggest the need for targeted policy interventions to enhance food security for this marginalized group. The study offers critical insights for policymakers, aid organizations, and stakeholders to develop effective strategies and policies that mitigate food insecurity among undocumented migrants, thereby contributing to achieving sustainable development goals by 2030.

## Introduction

The lethal virus, COVID-19, was first identified in December 2019 in Wuhan, China, prompting the World Health Organization (WHO) to declare a pandemic on March 11, 2020 [1]. Small, spreading chains evolved into larger chains of dissemination across multiple countries, resulting in extensive global transmission that affected all regions [2]. It is a pandemic of global health proportions that has the potential to trigger major socioeconomic, health, and food catastrophes worldwide [3]. The proportion of hungry individuals worldwide has increased significantly due to shutdowns and movement restrictions amid COVID-19, unemployment and underemployment have become common issues, significantly reducing people’s purchasing power [4, 5]. Several studies have also warned of the possibility of a catastrophic food crisis, particularly in nations currently undergoing financial turmoil, extreme weather events, or conflicts [5]. Since food is a fundamental necessity, food insecurity can result in famine, starvation, and malnutrition. It has been recorded that nearly all countries throughout the world have suffered in some way from food scarcity during this pandemic [6–8]. The COVID-19 pandemic has exacerbated persistent food insecurity issues in the UK and Washington, DC, affecting food and nutrition security, with the number of people experiencing hunger quadrupling since the crisis began [9, 10]. In South Africa, the COVID-19 pandemic has worsened the vulnerability of asylum seekers, refugees, and undocumented migrants, and they were systematically excluded from economic and hunger-alleviation schemes, exacerbating poverty and insecurity [11].

In terms of the number of migrants, Bangladeshis rank sixth in the world, with more than 13 million of their compatriots living outside the country. According to the Bureau of Manpower, Employment and Training (BMET) data, 617,209 Bangladeshi workers migrated to various nations worldwide in 2023, including the Gulf and other Arab and Southeast Asian countries [12]. Bangladeshis have long sought employment abroad, particularly in the oil-rich Gulf Cooperation Council (GCC) region [13]. A terrible consequence has been seen by migrant workers all around the world because of the COVID-19 pandemic. Migrant workers have borne the brunt of the pandemic’s effects, being stranded in their countries of destination or forced to flee, unemployed, and without access to social assistance. In Malaysia, for example, migrants constituted more than half of the workforce of many garment companies that went out of business, displacing thousands of people [14]. Undocumented migrants face increasing challenges in essential aspects of their lives. As a result, they had significant COVID-19 exposure, poor mental health, avoided seeking medical care, experienced income loss, experienced an acute food crisis, and experienced housing insecurity. In addition, they had a higher rate of COVID-19 morbidity and mortality [15, 16]. Undocumented migrants are less likely to seek health care or more likely to delay obtaining it due to concerns about their immigration status, such as fear of legal uncertainties, lack of awareness of entitlements, language and cultural barriers, or economic hardship [17]. Additionally, the lack of income increases the risk of homelessness [18].

The COVID-19 pandemic has had various negative socioeconomic consequences for Bangladeshi migrant workers, such as shrinking remittance flows, depleted savings, and job loss [19]. Migrant workers from Bangladesh in other countries face challenges such as joblessness, short working hours, exclusion, poor living conditions, social discrimination, and mental strain [20]. Many workers who have been allowed to stay in their host nations are isolated in filthy living quarters, psychologically troubled by worries about their jobs and family back home, and fearful of being deported to Bangladesh. Around 2,700 Bangladeshi people have died due to COVID-19 infection in various countries of the world till mid-2021, including large numbers in Gulf countries; others died in the U.S., U.K., Italy, and Saudi Arabia [21]. Undocumented Bangladeshi migrants, especially those residing in countries like Malaysia and Iraq, have faced severe hardships during the pandemic, excluded from formal employment and social assistance, resulting in extreme food insecurity [22]. Their lack of legal status often excludes them from formal employment, social welfare programs, and state-supported food assistance, leaving them particularly vulnerable to food scarcity [8]. In Malaysia, home to an estimated 1.2 to 3.5 million undocumented migrants [23], and Iraq, where economic instability and conflict further complicate access to resources, these individuals have experienced extreme levels of food insecurity. A comprehensive scoping review of 46 studies on international migrants during COVID-19 found that a vast majority of undocumented and precarious migrants experienced severe food insecurity, often resorting to reducing meal frequency, consuming low-quality diets, or relying on informal or charitable assistance, largely due to income loss and lack of access to state food aid linked to their documentation status [24]. Understanding the unique challenges faced by undocumented Bangladeshi migrants is crucial for addressing the broader issues of food insecurity and inequality during crises. Thus, this study aims to explore the extent and determinants of food insecurity and adaptive food-seeking behaviors due to the lack of access to safe and nutritious food among these migrants in Malaysia, Libya, and Iraq during the COVID-19 pandemic. By focusing on these two critical aspects, the researchers have set the following two objectives:

i. To explore the determinants of food insecurity among undocumented migrants in Malaysia, Libya, and Iraq during the COVID-19 pandemic.
ii. To understand the adaptive food-seeking behaviors adopted by undocumented migrants in Malaysia, Libya, and Iraq in response to food insecurity during the pandemic.

This research is essential in shedding light on the urgent need for inclusive policies that protect undocumented migrants, particularly in times of global crises. The findings will contribute to a deeper understanding of how Bangladeshi undocumented migrants navigate food insecurity, highlighting the socioeconomic disparities that require immediate attention to build a more equitable and resilient global food system. It also contributes to the achievement of Sustainable Development Goal 2, which seeks to end hunger and ensure access to food for all by 2030.

### Theoretical framework

This paper uses the adapted Sustainable Livelihoods Framework (SLF) combined with Social Exclusion Theory (SET) to examine food insecurity and food-acquisition practices among undocumented Bangladeshi migrants concerning COVID-19. This theoretical analysis theorizes the interplay between structural exclusion and agency within a legal environment of precarious legality and institutional marginalization.

The SLF, formulated by Chambers and Conway and subsequently formalized by the Department for International Development (DFID) [25], provides a comprehensive framework that can be used to connect external shocks, household assets, policy contexts, livelihood strategies, and outcomes. However, SLF does not sufficiently account for the legal and institutional barriers that constrain access to these livelihood assets. To help bridge this gap, the framework is modified by adding the SET Social Exclusion Theory (SET) that defines exclusion as a system-mass phenomenon of denied rights and access in economic, social, and political spheres [26, 27]. This incorporation reinforces the PIPs (Policies, Institutions and Processes) dimension of SLF by making explicit how being ‘undocumented’ works as a legal and institutional constraint that hinders migrant workers’ ability to access core services and protection [28]. In this light, the COVID-19 pandemic should be perceived not as a health crisis tout court but rather as a global system shock that led to mass unemployment, for the supply chain to be disrupted and food prices to soar [6, 25]. For undocumented immigrants, the shock increased with exclusion and migration policies preventing access to the government’s food supports, wage subsidies, and healthcare [28–30]. This policy-arisen food inaccessibility due to exclusionary PIPs led migrants to the actual inability to access formal assistance and public space, as ‘arrest or deportation’ remained a living fear of involvement [28, 31]. Financial capital was thus drained quickly—sometimes in as little as 15–20 days—through the loss of a job and wage theft [28]. However, social networks, important in terms of food sharing and remittances, were sensitive to covariate stress; many networks failed to offer support when everyone was affected [28]. Material assets, such as mobile phones and jewellery, were sold to purchase food, which undermined the long-term resilience [29, 32]. There was still underutilization of human capital due to legal status restricting formal labor market access [18, 28]. “The coping responses (increased high interest borrowing, sale of personal and family assets, or seeking help in informal, religious or social ways) were indicative of limited agency rather than purposive adaptability” [33, 34]. These were survivalist food searching acts and were associated with negative livelihood outcomes: reduced food consumption, hunger, and accumulated long-term debts [6, 28, 33]. These results are not congruent with SDG 2 and further perpetuate the spiral of structural vulnerability [35].

## Materials and methods

### Research approach and sample size

This study employed an Interpretative Phenomenological Approach (IPA) to understand the food insecurity and food-seeking behavior of undocumented Bangladeshi migrants in the host states. Because this approach focuses on how individuals perceive and comprehend their experiences and the environment in which they dwell [36], qualitative phenomenologists dwell in this environment. Consequently, the IPA approach is particularly well-suited for exploring the food insecurity and food-seeking behavior among undocumented migrants in host states [37].

The participant selection followed a snowball sampling technique. Since the researchers were based in rural areas where many people migrated abroad, a few of whom had no legal documentation during the COVID-19 pandemic, they first approached known individuals and then, through them, reached others who also lacked legal documents in the host countries. The study employed a purposive snowball technique to ensure the inclusion of participants with relevant experiences and insights into the research objectives. A total of 27 participants in in-depth interviews (IDIs) were conducted, and the sample size was determined by the principle of data saturation, a widely accepted standard in qualitative research. Data saturation was reached when no new themes or insights emerged from additional data collection, ensuring that the findings captured the depth and breadth of the participants’ experiences [38]. This rigorous snowball sampling strategy, combined with a focus on data saturation, enhances the validity and reliability of the findings, ensuring that they reflect the lived realities of vulnerable undocumented migrant communities.

### Data collection and instrument

The choice to employ a semi-structured interview method was made to gain a deeper insight into the practical aspects related to the research topics [39]. Indeed, a semi-structured interview approach is particularly suited for delving into specific details and obtaining in-depth information pertinent to the research questions [40]. At the outset of the study, five preliminary interviews were conducted at the field level to refine the questionnaire. The research team subsequently adjusted the interview questions to align with the context. Following the initial interview, the interview guidelines underwent revisions, a common practice within qualitative research methodology [41]. Five potential respondents declined to participate in the study, citing concerns about confidentiality despite our assurance of a high level of privacy. However, as part of the consent procedure, the participating respondents were informed that their involvement was voluntary and that their responses would remain confidential. The survey results would not disclose their identities, and all participants provided oral consent. By using Google Meet, Zoom, and WhatsApp platforms, we collected 27 in-depth interviews from irregular migrants who have worked without legal documents in Malaysia, Libya, and Iraq.

Two mobile devices recorded the discussions during the interviews, which lasted between 25 and 54 min. In some instances, written notes were taken because certain respondents were reluctant to be recorded. Subsequently, the recorded interviews were meticulously transcribed, and another team of researchers conducted a thorough review to ensure accuracy and precision in the transcription process.

### Positionality and reflexivity

All members of the research team were born in Bangladesh, and three are currently affiliated with European institutions. Our shared Bangladeshi background provided us with insider knowledge of cultural norms, migration practices, and community networks, which helped in building rapport and trust with participants. At the same time, the perspectives of team members based in Europe offered an additional outsider lens, shaped by broader academic and policy debates on migration. We acknowledge that our positionalities may have influenced the formulation of research questions, interactions during interviews (e.g., power dynamics with undocumented participants), and the interpretation of findings. To minimize potential bias, we engaged in continuous team discussions (Zoom, Google Meet), critically reflected on our assumptions, and cross-validated interpretations to ensure a balanced and rigorous analysis.

### Data analysis procedures: quality assurance, validity and reliability

In this study, NVivo 12 by QSR International was employed for data management and analysis, as it has proven highly valuable for analysing substantial volumes of text data, offering more in-depth and practical data analysis [38, 42]. This software was used to thematically code, classify, and organize transcripts, effectively handling a significant volume of data to enhance the reliability and validity of the results [43, 44].

The authors employed a thematic approach for coding the data and conducted data analysis using NVivo 12. Specifically, we imported all transcriptions into the software and used NVivo’s coding features to categorize the data systematically. Codes were developed based on the research questions and emergent themes derived from the data. This allowed us to maintain a structured approach to the analysis while remaining open to new insights. The data analysis followed the six-step process of Interpretative Phenomenological Analysis (IPA) as outlined by Smith, Folwers, and Larkin (2009), involving iterative reading, initial noting, development of emergent themes, clustering into superordinate themes, and interpretation of participants’ meaning-making [45].

## Results

This section presents a thematic qualitative analysis of findings derived from respondents using NVivo 12 software. The demographic profile of the participants is crucial for understanding their strengths and capabilities at work. As indicated in Table 1, the respondents’ ages range from 20 to 60 years, with 10 from Malaysia, 10 from Iraq, and 7 from Libya. Additionally, the majority of respondents lack formal education and are married, highlighting the socioeconomic challenges they face in their host countries.

**Table 1:** Respondents’ demographic profile

Category	Variable	*N*
Gender	Male 27	
Age	20–30	5
	30–40	10
	40–50	8
	50–60	4
Education	No formal education	3
	Under Primary	10
	Primary	5
	High School College	6 3
Marital status	Married	18
	Unmarried	9
Place of residence	Malaysia Iraq Libya	10 10 7

**Fig. 1:**
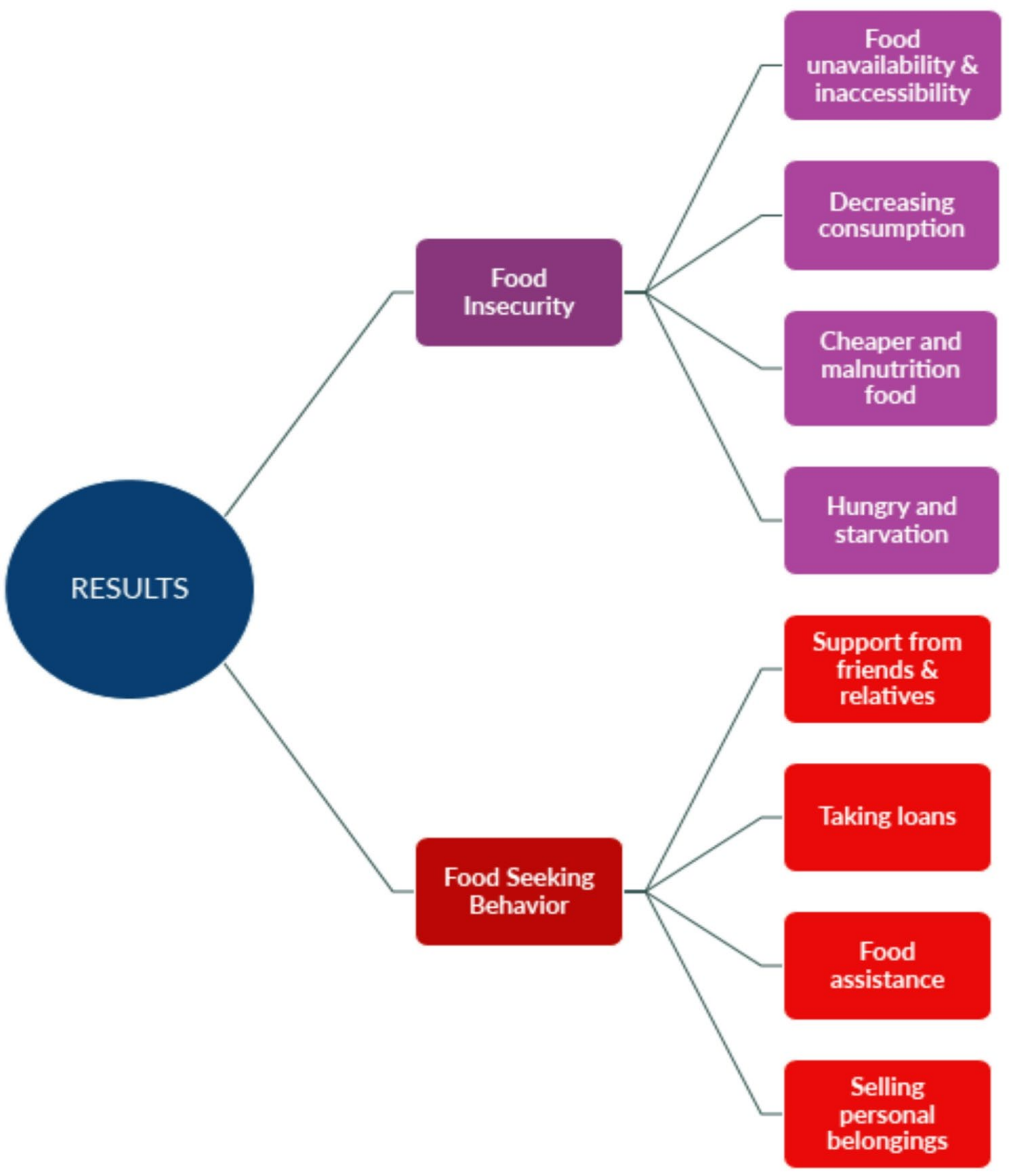
Food insecurity and food seeking behavior among undocumented Bangladeshi migrants

Our results and discussion are divided into two subsections, highlighting food insecurity and food-seeking behavior among undocumented migrants during the COVID-19 pandemic. Based on the NVivo analysis, we have demonstrated two themes: Food insecurity has four sub-themes, and food-seeking behavior has four sub-themes (see Fig. 1). Table 2 illustrates the themes and sub-themes derived from NVivo 12 software, and Fig. 1 explores the overall findings of this manuscript.

**Table 2:** Defined themes derived from the NVivo-12 analysis

Study Focus	Theme	Sub theme	Reference Code from NVivo-12	Descriptive Coding
Food Insecurity and Food-Seeking Behavior	Food insecurity	Food Unavailability & Inaccessibility	113	*“It was terrible because there’s nothing you can do. I ate only a small amount so as not to die. So that I can survive the next few days by eating something.”*
		Decrease consumption	95	*“I used to eat three times a day. Now we eat once. Even when I ate*,* ate very little.”*
		Cheap food	82	*“When I went to the shop*,* I looked for the less expensive food items. I used to buy lower price rice instead of a higher price of rice.”*
		Hungry & starvation	70	*“I lost my job during COVID-19*,* so I had to start skipping meals to have enough food for the next days. For this reason*,* mostly*,* I could spend my days struggling with hunger.”*
	Food Seeking Behavior	Friends and Relatives Support	82	*“At first*,* I was frugal in the food field. But later*,* my older brother would give me some money for food. That was a blessing for me.”*
		Taking loan	69	*“When I faced any crisis during COVID-19*,* then borrowed money from my childhood friends or relatives when I was in a food crisis.”*
		Food Assistance	50	*“One day*,* I saw through the window of my room some people in the street donating food to the poor and miserable people. I ran over there and got a 5–6 kg food bag.”*
		Selling Properties	48	*“But I had to sell my favorite mobile phone when I was out of work for 3 months. Selling it made my food less difficult.”*

### Food insecurity

One of the biggest challenges facing humanity in the 21 st Century is food insecurity during the COVID-19 pandemic. According to the report by the FAO, nearly a third of the world’s population, amounting to around 2.37 billion people, had insufficient access to food in 2020, an increase of around 320 million people from the preceding year [46]. This has been largely due to the COVID-19 crisis. This event has significantly disrupted human activities globally and posed a historic challenge to food security through widespread loss and disruption of job prospects. Specially third-world countries like Bangladeshi migrants who are in Malaysia and GCC have suffered the most [47, 48].

#### Food unavailability & inaccessibility

We identified that the majority of the Bangladeshi undocumented migrants are unable to guarantee access to food during COVID-19 due to the unavailability of basic food items in local neighborhood stores during lockdowns, and due to their undocumented status limiting accessibility to other retail stores. Some of the participants talked about visiting various shops and empty shelves; some said that they would not engage in bigger markets at all due to the fear of being detected. As an illustration, Respondent #12 noted:

“The food supply in the market was not adequate. When I went to the market to buy food, I found everything empty. Although I could not go to the big markets for undocumented status. But, before the lockdown, the food supply in these local markets was adequate.*”*

Access to food was also limited due to affordability. The loss of jobs, shortening of working hours, and theft of wages implied that not all participants had enough financial means to buy food, even when it was offered. The situation was also aggravated by sharp price inflation that caused households to restrict the quality and quantity of food they consumed. As Respondent #26 recalled:

*“Five months after the start of the epidemic*,* my company had to off for two months without paying. I had to suffer a lot to eat twice a day*. *I took low-quality food*,* as it is less expensive.”*

These results underscore the simultaneous presence of supply-side deficits, price shocks, and legal restrictions that negatively affected the capacity of undocumented migrants to obtain food to support their daily survival.

#### Decrease consumption

Traditional consumer consumption behavior has been altered because of the COVID-19 pandemic. Loss of jobs and increased food prices made most of the respondents turn to consuming less food, often rationing to make it through the lockdown period. The subjects reported a radical change where they used to eat three meals a day, to one or two meals a day. They did not have the resources to make sure sufficient food was available because they lost their employment, and were unable to earn money since lockdowns and spending an idle time in the room did not allow them to earn money, so they had to eat less food. Already, portions were being decreased- those who used to have two plates of rice, now had one. In this favour Respondent #7; felt that

*“At the beginning of COVID-19*,* I had a job*,* so I ate three meals a day without any worries. But since the lockdown started*,* I have been eating less*,* although I used to eat two plates of rice*,* but then I used to eat one plate. I did not eat on a full stomach. Because I sent all the salary home what I got. Since there was no work now*,* I never ate on a full stomach; I used to store food so that I would not have to suffer in the next days.”* after taking a long breath, he added. *“I lost weight*,* and my immunity became low. I always felt sick because of poor feeding.”*

We found out that food crisis in the market was a typical situation in the host countries where Bangladeshi migrants were. Where other people were concerned, reduced consumption did not merely concern rationing, but also availability. When the staples ran out of the local markets, the respondents spent days consuming the food of one kind in extremely minimal quantities. In addition, some respondents mentioned that they did not want to go outside due to police as they were not any legal documents. They could hardly afford three meals a day when they had no wages, and the increase in commodity prices corresponded to their impossibility to afford food.

*“One day I secretly went to the grocery store to buy some food items but did not find any rice*,* pulses*,* potatoes*,* sugar*,* or vegetables in the store. Then I went to another shop*,* but I got only 2 kg of rice and six-piece eggs. Thus*,* I survived by eating very few amounts of it for about ten days.”* (Respondent #21)

#### Cheap and malnutrition food

The undocumented migrants were acutely financially insecure during the pandemic: as the lack of permanent employment and income directly translated into food scarcity. This has resulted in affordability becoming the main factor affecting food decisions, with many people being forced to take cheap and in most cases unhealthy foods. Rather than buying new and healthy food, respondents said they had to use foods that were cheap yet compromised in quality: slightly stained or rotten vegetables, cracked eggs, and cheap dry foods such as biscuits, cakes, or bread. They knew they were risking their health by consuming such food, but low income did not give them much other choice. As one respondent explained:

*“When I went to buy food then always thought I had to purchase cheap food due to a shortage of money. So*,* I usually found slightly stained or rotten vegetables*,* cracked eggs*,* and dry food. I could buy these at a lower price.”* (Respondent #17)

A majority of the undocumented migrants have had a very pitiful financial crisis caused by COVID-19. That is the reason why they could not afford any nutritious food since the cost of nutritious food was too high compared to other foods. Due to this, the impact of hunger and malnutrition was felt most among the irregular migrants. In this instance Respondent # 9; a 30 year old migrant addressing as a brother told us the following:

“I was aware that consuming more nutritious food during the epidemic would be better for my health. However, the cost of such food was far too high for an undocumented immigrant like me to afford during the COVID-19 lockdown.”

Some of the respondents indicated that they had been consciously avoiding supermarkets which they deemed unaffordable and had been having to rely on local grocery stores or on open markets where the prices were relatively lower. One respondent explained:

*“Salary is off*,* so I used to buy food from local shops or local markets because everything is higher in the supermarket.” *(Respondent #14)

#### Hungry & starvation

At the time of the COVID-19 pandemic, undocumented migrants were left vulnerable due to decreased household incomes and supply chains while the pandemic exacerbated global food insecurity. The results of the study indicate that the undocumented Bangladeshi migrants in the host nations experienced serious hunger and in most instances, they experienced starvation. Precisely, the respondents stated that they could not venture out to buy food because they feared being arrested by the police, and the lack of income and employment further rendered them incapable of getting food. Consequently, persistent hunger, missed meals and long-term dietary deprivation during the lockdown period were widely reported. One of the participants was dwelling on how he had to make painful adjustments after he lost his job:

*“I lost my job during COVID-19*,* so I had to start skipping meals to have enough food for the next days. For this reason*,* mostly*,* I could spend my days struggling with hunger. After months of eating just once a day*,* I noticed my weight has fallen more than before.” *(Respondent #15)

It is also noted that the systematic challenges of undocumented migrants are that they have no access to institutional safety nets or law protection. Some of the respondents reported that their employers took advantage of their irregular status and did not pay them for months and unlike the documented workers they did not have access to any food or financial aids during the crisis. Though legal employees were provided by some companies with basic ration, illegal workers were not assisted in any way, which led to starvation and severe suffering.

*“My food and money are gone in the second lockdown. My company did not pay for the last two months. So how will I mitigate my hunger? Without salary*,* was it possible? Consequently*,* I had to experience without food for about two days*,* sometimes three days in a week. When I remember this*,* the fur on my body becomes erect. I thought I would die of food crisis.”* Said with tears eyes (Respondent #4)

### Food seeking behavior

We observed a cry for food among the Bangladeshi undocumented migrants as they worked there temporarily or contractually due to their undocumented status. So, without work and earnings, they had to face an extreme food crisis, forcing them to follow different food-seeking behaviours to survive in this pandemic situation. Our results showed that food support from friends and relatives, taking loans from home and abroad to ensure food, selling properties to cover daily necessities, and food assistance from various sources were the main food-seeking behaviours adopted by Bangladeshi undocumented migrants to adapt to the situation during this pandemic.

#### Friends and relatives support

The COVID-19 pandemic had a devastating impact on the employment of undocumented migrants because numerous people were dismissed during lockdowns when the number of company activities reduced. Unlike the legal ones who were at times left behind on half-time work, illegal workers were the first to lose their job. This abrupt unemployment brought on acute economic distress, specifically in the food insecurity. Under these conditions, friendship and relatives became an important informal support system. One of the respondents emphasized the significance of family relations, as he explained how his brother with legal documents was able to provide him with financial support at this period when it was so critically important:

*“We two brothers live in Malaysia. My older brother had all the legal documents*,* but I had not. So*,* he could work as a half-duty in their company. But since the first lockdown*,* I have been without work for about 4 months*,* so at first*,* I was frugal in the food field. But later*,* my older brother would give me some money for food. That was a blessing for me.” *(Respondent #11)

Although the assistance of the family was also important, it was not always available. Close relatives were not near in the same country by many of the Bangladeshi migrants. Friendships, which in many occasions were created at work or during living arrangements, were a critical safety net in such cases. Sharing of meals and pooling of resources also helped avoid acute starvation in migrants, and such cross-national solidarity was also prominent as migrants with other nationalities helped one another out of the crisis. As one of the participants explained:

*“We were Indians*,* Pakistanis*,* Somalis*,* and some Nepalese working together in the same company and living in the same house. Due to lockdown*,* my company permanently cut me off because I joined this company temporarily as I had no legal documents. My friends used to cover all our food expenses*,* but I did not have to pay any expenses*. *It is a matter of luck to have such friends abroad.”*

Without institutional assistance, without document, migrants were extensively dependent on these informal safety nets to survive. This support was however unequally distributed and could only be offered by the availability of family members or reliable friendships leaving many still highly vulnerable to hunger and deprivation.

#### Taking loan

Migrations of Bangladesh are frequently linked with enormous financial expenses since a large number of migrants go to other countries with loans or selling their property. After they are employed, their major role is to send remittances home and settle debts leaving them with minimal savings. This cycle was interrupted by the COVID-19 pandemic because the sudden loss of jobs and extended lockdowns left undocumented migrants without income and, therefore, with no opportunity to cover everyday costs. Within a few days of lockdown, a large number of people were left moneyless, and they and their families had to sustain themselves using new loans. Migrants were in some instances borrowing money through their family members in Bangladesh to support them in foreign countries. One respondent described:

*“When my father heard that I had been out of work for two months and suffered from a food crisis*,* my father gave me TK 1 lakh on interest from a Money Lender. God knows what would have happened to me if I had not received the money*.” (Respondent #14)

In addition to institutional or moneylender loans, a good number of undocumented migrants resorted to informal borrowing among family members, friends and acquaintances to meet basic needs like food. Such borrowing tended to cross borders and represented the power of transnational networks of support between migrant communities. As an illustration, one of the participants elaborated:

*“When I faced any crisis during COVID-19*,* then borrowed money from my childhood friends or relatives when I was in a food crisis. At first*,* I took 10 thousand BDT from my uncle. After some days*,* this money ran out*,* and again*,* some other necessities*,* including food*,* appeared. Then I borrowed 35*,*000 BDT from my childhood friend*,* who lived in Saudi Arabia though I was in Malaysia. That’s how I had to deal with this pandemic.”*

Nevertheless, reliance on expensive lending by moneylenders and NGOs only made them even more financially vulnerable, a cycle of debt that could linger on long after the crisis. Survival in the lockdown was achieved at a price of long term indebtedness by many, further complicating the pre existing vulnerabilities linked to undocumented migration.

#### Food assistance

The food insecurity of the undocumented migrants was at severe levels at the time of the COVID-19 pandemic, driving a significant number of undocumented migrants into an extremely precarious state. Although, some employers and organizations tried to alleviate the crisis through food aid, this guidance was not universal and even uniform. Some of the respondents stated that even being undocumented, their employers did provide them with daily food supply in the time of the lockdown, which allowed them to temporarily survive through the crisis despite the absence of salaries. As one participant noted:

*“My company was very supportive during induced lockdown because all the activities of our company shut down during COVID-19 induced lockdown. But it provided necessary foods but not pay salary.” *(Respondent #25)

In addition to the company initiatives, there were also charitable aid of volunteers, religious organizations and personal donations to which migrants turned. Finding food aid was a survival tactic by some, to the extent of walking the streets to find donations. One respondent described:

*“I often roamed beside the street to get food donations.” *(Respondent #18)

Religious institutions including mosques and temples turned into essential safety nets, with religious people supplying food to the poor and vulnerable, during lockdown. To the undocumented migrants who had no income or houses, these institutions provided food and temporary safe haven. One of them said:

*“About three or four months after the Coronavirus started*,* all my money was spent. Because I couldn’t do any work. To overcome the food crisis in this situation*,* I was forced to stay in local mosques and survive on the charities provided by the mosques.”* (Respondent #8).

Conversely, the research revealed that there are organizations and volunteer groups who volunteer to supply food to the needy individuals. Bangladeshi migrant workers were among those who had encountered the problem of food crisis, and had lost employment. One of the participants explains this state as;

*“One day*,* I saw through the window of my room some people in the street donating food to the poor and miserable people. I ran over there and got a 5–6 kg food bag. After coming back*,* I informed all my roommates*,* then they also got such food assistance like me. After that*,* I looked through the window because someone would come again like that.” *(Respondent #2)

#### Selling personal belongings

The research revealed that a lot of migrants were planning to go back to the country because they did not have legal papers. Since in case the police apprehend them, they will be required to pay huge fines and be harassed. This is why a lot of illegal immigrants chose to send money home to purchase gold ornaments and other items to their mothers, wives, and sisters. However, COVID-19 ruined their plan and made it their curse. Consequently, endless misery and despair comes in their lives, and an extreme food crisis had to be endured. In this case, they sell them and use them to earn their livelihood. One of the respondents told of his difficult experience

*“I bought two gold necklaces for my wife and mother and some cosmetics for my younger siblings to come home. But I could not go home from lockdown. At first*,* I sold my wife’s necklace; I thought the epidemic would go away very soon. But when that did not happen*,* I was also forced to sell my mother’s necklace because I had been out of work for about 6 months. If I didn’t sell them*,* I would die without eating.” *(Respondent #19)

Besides ornaments, some of the respondents have sold their best technological gadgets, their mobile phones. To most of the migrants, a mobile phone is their only personal possession and also their means of communication with family back home. However, when hunger struck even these necessities were compromised in the name of getting food. As one respondent explained:

*“I had two mobiles*,* one was an Android smart mobile*,* and the other was a button phone (Analog mobile). But I had to sell my favorite mobile phone when I was out of work for 3 months. Selling it made my food less difficult. My roommate*,* the Bengali friend*,* is also trying to sell mobile but is not getting customers.”* (Respondent #3)

## Discussions

Aligning with the global data, the present study found that undocumented migrants were more vulnerable to food insecurity during COVID-19 [49, 50]. Despite the presence of food in national supply chains, undocumented migrants often could not convert this into real access. Affordability further restricted access. Job loss, unpaid wages, and wage theft eroded financial assets, while price inflation made staple foods unaffordable. In the Middle East, thousands of Bangladeshi migrant workers are spending days in utter crisis abroad as they are losing jobs, accessing food in limited numbers [51]. Similar dynamics have been reported globally; migrants in multiple host countries reported inability to purchase adequate food due to rising prices and lost income [49, 52]. They also face demand and supply-side obstacles, including high food prices and poverty, as well as supply-side obstacles, such as empty shelves within local markets. Previous study showed that COVID-19 led to price distortions and shortages in urban food markets and disrupted food supply chains in India, leading to a 10% drop in product availability for vegetables, fruits, and edible oils [53]. Thus, food insecurity in this context is not merely about scarcity but about structurally mediated inaccessibility [54].

We found a drastic change in the frequency and amount of meals consumed, which was reported by the respondents, with many changing their meals to one or two per day, as mentioned in an earlier study that migrant worker consumes less due to food insecurity [55]. This is because prices of aggregate food like meat, dairy, sugar, and vegetable oil were also raised drastically after the lockdowns [33, 56]. There was much mention of rationing of foods over long durations, even at the expense of health, weight, and immunity. These coping strategies mirror global trends during COVID-19, where households facing economic shocks reduced consumption, variety, and dietary quality [57]. Specifically, meal skipping and rationing have been the most commonly reported strategies among migrants [49].

The study also reveals that the inability to get consistent employment and income meant that migrants had to focus on affordability rather than quality food. This led to the consumption of low-quality, usually unhealthy types of food, including slightly spoiled vegetables, cracked eggs, and low-cost dry products. Such practices reflect a common survival strategy among food-insecure populations, prioritizing caloric sufficiency over nutritional adequacy [57]. This concurs with the adapted Sustainable Livelihoods Framework (SLF) in which financial and physical resources were quickly exhausted, leaving migrants with a limited agency and less capacity to obtain sufficient food [25]. These trends reflect previous research, which recorded hunger and starvation in irregular migrants in crises because of inadequate availability and access to food [58]. The result is a decrease in dietary diversity, which increases malnutrition and health susceptibility risks.

Perhaps the most severe manifestation of food insecurity among our respondents was hunger and sometimes prolonged starvation. Several migrants reported skipping meals for days, experiencing dizziness, weight loss, and persistent hunger. These conditions reflect the compounding effects of job loss, employer exploitation, exclusion from relief measures, and fear of arrest. Research indicates that COVID-19 lockdowns severely impacted food security among informal migrant workers in Bangladesh and India, leading to hunger, starvation, and malnutrition [28]. The undocumented migrants were left out of the formal support mechanisms, unlike the documented workers who were occasionally issued with rudimentary rations or half salaries. The non-payment of wages by employers further undermined vulnerability, which is in line with the existing literature on exploiting irregular workers due to emergencies [59]. The threat of arrest, unemployment, and loss of wages meant that migrants could not use formal or alternative sources of food, and this is what the Social Exclusion Theory defines as deprivation instigated by the institutions. Such circumstances aggravated the circumstances of these undocumented Bangladeshi immigrants in the host countries, and this brought a catastrophic impact on world hunger and poverty, particularly to the undocumented migrants [60].

The results demonstrate the adaptive coping mechanisms of undocumented Bangladeshi migrants to extreme food insecurity in the COVID-19 pandemic, which underscores the resilience and vulnerability of the population. In line with the adapted Sustainable Livelihoods Framework (SLF), migrants’ agency is limited by relying on relatives and friends, borrowing, food aid, and selling personal resources, which is reflected in their coping behaviors.

In our study, Friend and family support was identified as one of the main informal safety nets as a coping strategy, especially when formal support was not available as a result of undocumented status. This aligns with findings from other contexts: undocumented migrants often rely heavily on informal social capital when formal systems exclude them. For example [61], reports that service providers observed that undocumented immigrants during COVID-19 usually depended on tight-knit community networks instead of government assistance. A previous study showed that Informal social support from family and friends is a key form of support for migrant domestic workers [62].

Another vital coping mechanism was borrowing money, either within Bangladesh or outside, from relatives, friends, or local moneylenders. Migrants who borrowed a significant amount of money from various organizations and individuals to address the COVID-19 situation [63]. As observed in previous research, borrowing a loan was a drastic coping mechanism to the food crisis in the COVID-19 pandemic [64] [65],. Although this form of loans allowed survival in the short run, it ultimately leads to long-term debt, which is long-established within the scope of migrant precarity [66]. These results indicate that COVID-19 produced short-term food insecurity and increased the structural financial vulnerabilities that will continue even after the pandemic.

Employer and charitable organization food and religious food helped provide an added buffer against starvation. However, this was only occasional and inadequate, which demonstrates the gaps in official and semi-formal safety nets of undocumented populations. Depending on donations or temporary shelters on religious premises, with the help of roaming, it proves that the migrants went to the last extremes to find food. This behavior echoes global patterns: marginalized migrants often access third-sector or informal aid when state support is unreachable [67]. In many localities, civil society and religious charities became critical backstops during COVID-19 when government programs excluded undocumented populations [32]. Regular migrant workers receive this privilege, but irregular migrant workers were excluded from Covid-19 relief efforts due to a lack of legal documents, exacerbating their vulnerabilities [68].

When all else failed, many migrants sold personal belongings—jewelry, mobile phones, household items—to generate cash for food. One respondent sold his wife’s and mother’s necklaces; another sold his own smartphone, knowing that reduced connectivity risked isolation but was outweighed by immediate need. Pakistani migrant workers in Malaysia sold personal belongings to generate cash for food during the COVID-19 lockdowns [69]. This pattern reflects the idea of disinvestment of assets as a coping strategy in livelihoods literature: reducing capital to maintain consumption. Under ASLF, this is a signal that migrants have exhausted more sustainable strategies and are entering crisis mode.

This paper connects SLF and SET by highlighting the functioning of undocumented status as a structural determinant of health. Lack of food aid, healthcare services, and wage protection added to malnutrition, low immunity, and increased psychological stress. The stories of the migrants of Hunger, starvation, selling of representational objects of wedding jewelry, etc., not only deprivation of material resources but also of social disgrace and loss of dignity, which are the key dimensions of SET. These results support the fact that exclusionary migration policies not only undermine the livelihood of undocumented workers but also directly oppose the global obligations under Sustainable Development Goal 2 (Zero Hunger).

## Conclusion and policy implications

The effect of the COVID-19 pandemic has had very significant implications for the lives of Bangladeshi migrants, with undocumented laborers in Malaysia, Libya, and Iraq bearing the brunt. This qualitative research paper has noted that lockdowns and unemployment related to the pandemic caused food insecurity to affect the whole population, causing migrants to eat less, rely on low-nutritional foods or unsafe ones, and, in certain instances, prolonged hunger and malnutrition. Strategies that were reported to be used by participants include borrowing money, food sharing between peers, and seeking help from religious institutions, volunteers, or informal community networks. Most were left out of the basic protection without proper support from employers, host governments, or even formal aid agencies. These data indicate that food insecurity in undocumented migrants is not a humanitarian issue. Still, it is also a health concern, and its effects are on the nutritional status, immune system, and crisis resilience. Coming out of official safety nets can contribute to inequalities and weaken group preparedness to pandemics. To address these gaps, context-sensitive and inclusive policy interventions are needed to acknowledge the interrelations between migrant health and other population health outcomes. In the case of the Bangladeshi government, more powerful protection mechanisms can be helpful. Multilingual hotlines may be offered in the embassies and consulates to communicate more effectively in an emergency. So, a contingency fund could be used to provide quick cash or food aid. Improving pre-departure orientation with crisis preparedness, awareness of rights, and available sources of support can also increase the resilience of migrants. The organizations that turned out to be crucial first responders during the pandemic are community-based and religious organizations. Financial and logistical assistance and confidential referral training might help these organizations better connect with undocumented migrants. The role of international organizations and donors like International Organization for Migration (IOM), World Food Programme (WFP), and World Health Organization (WHO) may be significant as they will support the idea of inclusive humanitarian policies, fund pilot projects (e.g., voucher schemes or community-based food banks), and initiate bilateral collaboration between Bangladesh and a host country regarding emergency preparedness and response. These measures taken together can not only lead to the reduction of food insecurity among undocumented migrants but also to the social cohesion and stabilization of the labour market and achievement of the Sustainable Development Goals. Although this study has the limitation of a small qualitative sample, the information that it has produced can form a valuable base for future research. Predominantly, larger and mixed-method research would be helpful in determining the prevalence of food insecurity among migrants and evaluating the effectiveness of inclusive policy responses.

## Data Availability

The data used in this study are available upon reasonable request from the corresponding author, whose contact information is provided on the first page of the manuscript.
